# Human Coronavirus 229E Uses Clathrin-Mediated Endocytosis as a Route of Entry in Huh-7 Cells

**DOI:** 10.3390/biom14101232

**Published:** 2024-09-29

**Authors:** Sabina Andreu, Inés Ripa, José Antonio López-Guerrero, Raquel Bello-Morales

**Affiliations:** 1 Departamento de Biología Molecular, Universidad Autónoma de Madrid, 28049 Madrid, Spain; 2 Centro de Biología Molecular Severo Ochoa (Consejo Superior de Investigaciones Científicas), 28049 Madrid, Spain

**Keywords:** coronavirus, clathrin, viral entry, endosomes, HCoV-229E

## Abstract

Human coronavirus 229E (HCoV-229E) is an endemic coronavirus responsible for approximately one-third of “common cold” cases. To infect target cells, HCoV-229E first binds to its receptor on the cell surface and then can follow different pathways, entering by direct fusion or by taking advantage of host cell mechanisms such as endocytosis. Based on the role of clathrin, the process can be classified into clathrin-dependent or -independent endocytosis. This study characterizes the role of clathrin-mediated endocytosis (CME) in HCoV-229E infection of the human hepatoma cell line Huh-7. Using specific CME inhibitory drugs, we demonstrated that blocking CME significantly reduces HCoV-229E infection. Additionally, CRISPR/Cas9-mediated knockout of the µ subunit of adaptor protein complex 2 (AP-2) further corroborated the role of CME, as KOs showed over a 50% reduction in viral infection. AP-2 plays an important role in clathrin recruitment and the maturation of clathrin-coated vesicles. Our study also confirmed that in Huh-7 cells, HCoV-229E requires endosomal acidification for successful entry, as viral entry decreased when treated with lysomotropic agents. Furthermore, the colocalization of HCoV-229E with early endosome antigen 1 (EEA-1), only present in early endosomes, suggested that the virus uses an endosomal route for entry. These findings highlight, for the first time, the role of CME in HCoV-229E infection and confirm previous data of the use of the endosomal route at a low pH in the experimental cell model Huh-7. Our results provide new insights into the mechanisms of entry of HCoV-229E and provide a new basis for the development of targeted antiviral therapies.

## 1. Introduction

Human coronavirus 229E (HCoV-229E) is an endemic CoV responsible for approximately 20–30% of “common cold” cases in adults [1]. This enveloped virus belongs to the genus *Alphacoronavirus* (α), and, to enter cells, it uses human aminopeptidase N (APN), also known as CD13, a surface metalloprotease present on the membranes of host cells as a receptor [2], which is ubiquitously distributed in mammalian tissues such as the liver, lung, intestine, kidney or central nervous system [3].

A numerous variety of entry routes have been described for CoVs, with endocytosis being one of them [4]. Endocytosis is a cellular process by which external substances such as solutes, fluids, plasma membrane components and pathogens are engulfed into the cytoplasm through the inward folding of the cell membrane [4]. In addition to contributing to nutrient and ligand uptake, cell signaling and adhesion, among other functions, this process can be exploited by some viruses to enter and infect cells [5,6,7]. Depending on the role of clathrin, endocytosis can be classified into clathrin-dependent/mediated (CME) or clathrin-independent (CIE).

Focusing on HCoV-229E, after binding to its receptor APN, it uses host cellular proteases trypsin, cathepsin L and transmembrane protease, serine 2 (TMPRSS2) to complete spike (S) protein-mediated fusion activation [8]. Binding to the receptor triggers the fusion between viral and cell membranes, all in a low-pH environment, leading virions to enter, following an endosomal/lysosomal route [9,10]. However, the detailed endosomal pathway of how virions reach the lysosome has not yet been fully described, and it varies among different tissues [9,11]. In addition, previous studies report that HCoV-229E utilizes a caveolae-dependent endocytic pathway for entry [12], but no research has been performed on the role of CME in the entry of this virus. Nonetheless, other coronaviruses such as SARS-CoV-1, SARS-CoV-2, murine hepatitis virus (MHV) or feline infectious peritonitis virus (FIPV) have been reported to use the CME pathway [13,14,15,16]. More precisely, another CoV from the α-genus, HCoV-NL63, has been described to use the pH-sensitive CME route [10].

Here, we demonstrate, for the first time, the role of CME in the entry of HCoV-229E into the human hepatoma cell line Huh-7, the experimental model chosen, as it has been previously reported to be highly susceptible to HCoV-229E infection [17]. In addition, Huh-7 cells are polarized epithelial cells that highly express APN, and the entry and release of many HCoVs, including HCoV-229E, have been previously associated with the apical cells [18,19]. Treatment with CME-blocking drugs results in significant decreases in infection. In addition, knockout, by the CRISPR/Cas9 strategy, of the µ subunit of adaptor AP-2, a complex essential for clathrin recruitment, led to an inhibition of viral entry. This adaptor is the second most abundant component of clathrin vesicles, and without it, mature vesicles fail to form and the route stops [20]. In addition, HCoV-229E also requires a low-pH endosomal environment to enter Huh-7 cells, as seen previously in other cell lines [21,22]. These results provide evidence that the absence of CME reduces HCoV-229E infection and that this endosomal route, following acidification, is used by this virus, which can be applied to design antiviral strategies that target this pathway.

## 2. Materials and Methods

### 2.1. Cell Cultures

A Huh-7 cell line, originated from human neuroblastoma cells [23], was generously provided by Dr. Sonia Zúñiga (CNB-CSIC, Madrid, Spain). The cells were maintained in low-glucose Dulbecco’s modified Eagle medium (DMEM) (Life Technologies, Paisley, UK) supplemented with 10% fetal bovine serum (FBS), glutamine (2 mM), penicillin (50 U/mL) and streptomycin (50 μg/mL) (Gibco, Waltham, MA, USA) at 37 °C in a humidified atmosphere of 5% CO_2_. The absence of mycoplasma was confirmed by using the PCR Mycoplasma Detection kit (Takara Bio, San José, CA, USA).

### 2.2. Viruses

Recombinant strain HCoV-229E expressing the GFP reporter protein (HCoV-229E-GFP) was kindly provided by Dr. Volker Thiel (University of Bern). This virus was propagated in Huh-7 cells for 3 days at 33 °C with 5% CO_2_. The infectious titer of the viral stocks was determined, according to the Reed and Muench [24] formula, on Huh-7 cell monolayers by the endpoint dilution assay, calculating the 50% tissue culture infectious dose per ml (TCID_50_/mL), as further described.

### 2.3. Antibodies and Reagents

Chlorpromazine (C8138), dynasore (D7693) and pitstop 2 (SML1169) were obtained from Sigma-Aldrich (St. Louis, MO, USA). Mowiol was purchased from Calbiochem (Merck Chemicals, Darmstadt, Germany), DAPI was obtained from Thermofisher (Waltham, MA, USA) and human transferrin (Tf) CF^®^543 and CF^®^555-Labeled Dye Dextran 10,000 MW conjugates were obtained from Biotium (Fremont, CA, USA). All the drugs used were dissolved in 0.1% *v*/*v* dimethyl sulfoxide (DMSO), except for chlorpromazine and Tf, which were dissolved in sterile distilled water.

The primary antibodies used were mouse monoclonal anti-GFP (11814460001; Roche, Basel, Switzerland), mouse monoclonal anti-β-actin peroxidase (A3854, Sigma) and rabbit monoclonal anti-AP2M1 (ab75995; abcam, Cambridge, UK). Horseradish peroxidase conjugate (HRP) secondary anti-IgG antibodies were purchased from MilliporeSigma (Darmstadt, Germany).

### 2.4. Analysis of Cell Viability

The cytotoxicity of chlorpromazine, dynasore and pitstop 2 in Huh-7 cells was analyzed by the MTT [3-(4,5-dimethylthiazol-2-yl)-2,5-diphenyltetrazolium bromide] assay, using a Cell Titer 96^®^ Non-Radioactive Cell Proliferation Assay Kit (Promega, Madison, WI, USA). Non-confluent monolayers of Huh-7 cells plated in 96-well tissue culture dishes and cultured in DMEM supplemented with 5% FBS were incubated for 24 h with different concentrations, between 0 and 100 μM, of each compound. Four replicates were carried out for each concentration. Then, the cells were incubated as indicated by the manufacturer of the kit, and the resulting colored solution was quantified using the scanning multiwell spectrophotometer iMarkTM Microplate Reader (BioRad, Hercules, CA, USA), measuring the absorbance at 595 nm. The readouts obtained from the MTT assay were further normalized to the value of untreated cells, where the viability value was set to 100%.

### 2.5. Endocytosis Assay

Cells grown on 24-well plates on round coverslips were treated for 1 h with either 10 μM chlorpromazine, 100 μM dynasore or 50 μM pitstop 2 at 37 °C and then maintained for 30 min on ice with human transferrin conjugate Tf CF^®^543 (5 μg/mL) or CF^®^555 Labeled Dye Dextran 10,000 MW (5 μg/mL). All cells were further incubated for 5 or 10 min, respectively, at 37 °C in a humidified 5% CO_2_ atmosphere to allow the internalization of Tf or the dextran conjugate. Finally, the cells were washed with PBS and then fixed for immunofluorescence microscopy.

### 2.6. Treatment with Endosomal Acidification Inhibitors

Huh-7 cells cultured in 24-well plates were treated with 5, 10 or 20 mM NH_4_Cl for 1 h and then infected with HCoV-229E-GFP at an m.o.i. of 3. After 1 h of incubation at 33 °C in the presence of NH_4_Cl, the cells were washed with PBS and left for 3 h in the presence of the compound. Finally, the compound was removed, and the cells were maintained in the culture medium until 20 h p.i., where they were collected for flow cytometry and immunofluorescence analysis. In some assays, the cells were treated with NH_4_Cl from 1 h before infection to 6 h p.i. (short times) or from 6 h to 24 h p.i., with samples collected at 24 h p.i. for flow cytometry.

### 2.7. Immunofluorescence Microscopy

Cells grown on 24-well plates on round coverslips were fixed in 4% paraformaldehyde (PFA) for 15 min and rinsed with PBS. The cells were then permeabilized with 0.2% Triton X-100, rinsed and incubated for 30 min at room temperature (RT) with 3% bovine serum albumin in PBS (blocking buffer). For a labeled immunofluorescence analysis, the coverslips were incubated in a wet chamber and the nuclei were stained with DAPI for 10 min. After thorough washing, the coverslips were mounted on a slide using mounting media (Mowiol). Images were obtained using a LSM 710 Inverted Confocal Microscope (Zeiss, Vienna, Austria) equipped with an Argon laser and a He/Ne 633 nm laser. The pinhole size was set to 1 AU. Processing of the confocal images was performed using the Fiji-ImageJ software (version Image J 1.53c) [25].

### 2.8. Flow Cytometry

To perform fluorescence-activated cell sorting (FACS) analysis, the cells were dissociated by 3 min incubation with 0.05% trypsin/0.1% EDTA (Invitrogen, Waltham, MA, USA) at RT and washed and fixed with 1%-PFA-1% FBS in PBS for 15 min. Finally, the cells were rinsed and resuspended in PBS. The cells were analyzed using a FACSCalibur Flow Cytometer from BD (Franklin Lakes, NJ, USA). Data were processed with the FlowJo software (BD, version 10.6.2).

### 2.9. Endpoint Dilution Assay

Titration of HCoV-229E from infected samples was performed following the endpoint dilution assay, as previously described [26]. Confluent monolayers of Huh-7 cells were plated in 96-well tissue culture dishes and cultured in DMEM supplemented with 10% FBS. Serial dilutions (10^−1^ to 10^−9^) of samples previously infected with HCoV-229E were prepared and inoculated onto four replicate cell cultures. The cells were then incubated at 33 °C in a humidified atmosphere containing 5% CO_2_ for 48 h. Finally, the 50% tissue culture infectious dose (TCID_50_) per ml was determined, considering the final dilution that showed a cytopathic effect, and calculated using the Reed and Muench method [24].

### 2.10. Western Blot Analysis

The cells were lysed using the radioimmunoprecipitation assay (RIPA) buffer mixed with a protease inhibitor cocktail from Roche (Basel, Switzerland), and the total protein load was quantified by a Bradford assay (Bio-Rad Laboratories, Inc. Hercules, CA, USA). Equalized protein samples were separated by SDS-PAGE in 10% acrylamide gels under non-reducing conditions and later transferred onto Merck Millipore Immobilon-P membranes. The membranes were blocked for 30 min in 5% non-fat dry milk at RT, incubated overnight at 4 °C with the appropriate primary antibodies (anti-GFP, 1:1000; anti-AP2M1, 1:1000), washed with 0.05% Tween 20 in PBS and incubated with HRP-conjugated secondary antibodies for 1 h at RT. The anti-β-actin-peroxidase antibody (1:50,000) was directly incubated for 1 h at RT. After extensive washing, the membranes were finally incubated with an enhanced chemiluminescence Western blotting kit with ECL Western Blotting Detection Reagent (GE. Healthcare, Chicago, IL, USA) to visualize the protein band. The intensity of the immunoblot bands was analyzed using Fiji-ImageJ software (version Image J 1.53c).

### 2.11. Generation of AP2M1 Knockout Cell Lines

Alt-R CRISPR-Cas9 crRNAs were mixed with Alt-R CRISPR-Cas9 tracrRNA and heated at 95 °C for 5 min. crRNA:tracrRNA duplexes were incubated with Alt-R S.p. HiFi Cas9 Nuclease V3 to assemble the ribonucleoprotein (RNP). For RNP complex transfection, Huh-7 cells were electroporated (Nepa21, Sonidel) using the Alt-R Cas9 Electroporation Enhancer. The settings were (1) poring pulse: voltage 150 V, pulse length: 5 ms, pulse interval: 50 ms, no. pulses: 2, decay rate: 10% and polarity: + and (2) transfer pulse: voltage 20 V, pulse length: 50 ms, pulse interval: 50 ms, no. pulses: 5, decay rate: 40% and polarity: ±. Alt-R CRISPR-Cas9 system components were purchased from Integrated DNA Technologies (IDT). Finally, the cells were plated in 12-well tissue culture plates and maintained for 72 h in the same medium.

Subsequently, transfected cells were cultured in a 96-well plate at a density of 5 cells/well to grow single cell clones. Finally, the AP2M1 deficiency of KO cells was tested by immunoblotting. Primer design and RNP transfection were performed by the Transgenesis Core Facility at the Centro Nacional de Biotecnología-CNB (CSIC, Madrid, Spain). The gRNA for the KO of the human AP2M1 gene used was GATGTCATCTCGGTAGACTCGGGP [27], with the PAM sequence underlined.

### 2.12. Statistical Analysis and Quantification of Fluorescence

The results obtained from the experiments were analyzed using Prism software v8.0.1 (GraphPad software, Inc., San Diego, CA, USA). The data were subjected to Mann–Whitney U-tests to determine significant differences between groups, and a *p*-value of <0.05 was considered statistically significant. For fluorescence intensity quantifications, various regions of interest (ROIs) were measured from groups of 20 cells with 3 areas of each image to normalize for the cell number and background intensity. The Fiji-ImageJ (version Image J 1.53c) software was used to quantify the fluorescence intensity for the 555 channel. The images were transformed to 8-bit grayscale, and the fluorescence intensity was analyzed using the particle analysis function after correcting the background, as previously described [28].

## 3. Results

### 3.1. Targeted Inhibition of CME Using Chemical Inhibitors

The first approach followed to study CME was to block this pathway by using appropriate chemical inhibitors that inhibit this route by different mechanisms. For instance, chlorpromazine prevents the assembly and disassembly of clathrin networks on cell surfaces and on endosomes [29], dynasore inhibits the fission of clathrin-coated pits by blocking the GTPase dynamin and pitstop 2 interferes with the interaction between clathrin and adaptor proteins essential for clathrin-coated pit (CCP) formation [30].

**Figure 1:**
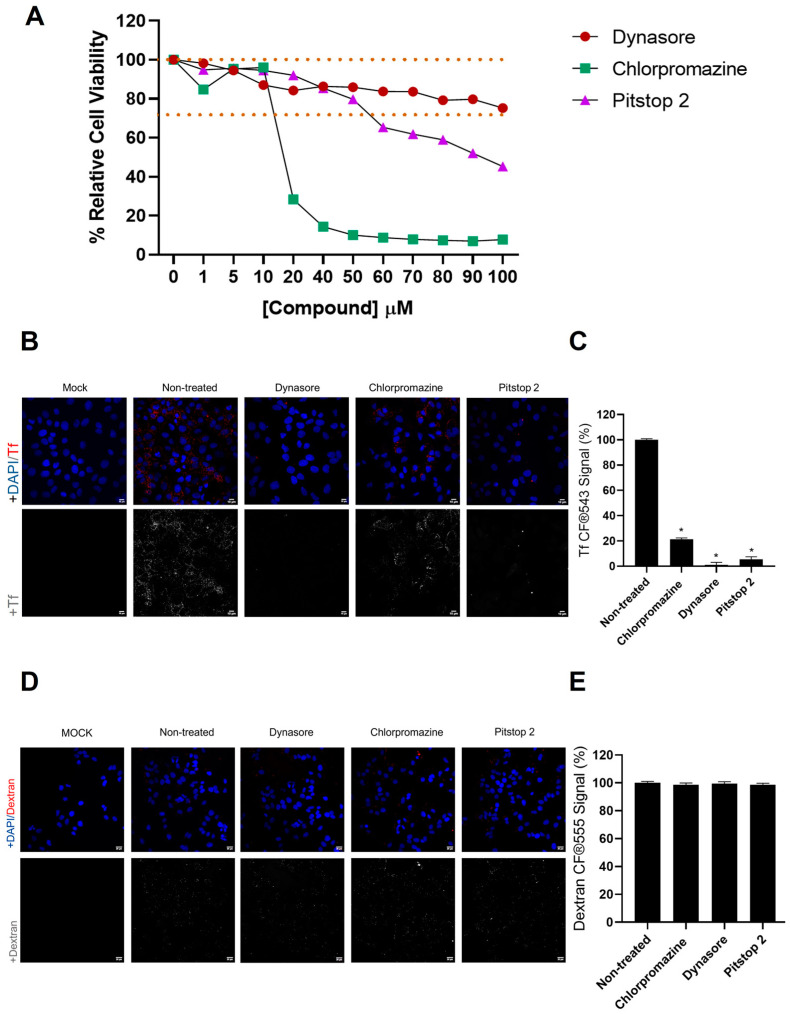
Chemical inhibitors of CME block conjugate transferrin but not conjugate dextran internalization at non-cytotoxic doses. (**A**) Cell viability of Huh-7 cells treated with chlorpromazine, dynasore or pitstop 2 for 24 h. Viability was measured by a MTT assay and calculated as the percentage of cell viability compared to untreated cells; the columns represent the mean percentage of relative cellular viability ± S.D. (*n* = 4) after drug exposure. The dotted lines mark the area in which values are considered non cytotoxic. (**B**) The uptake of transferrin in Huh-7 cells is blocked by CME inhibitors. Cells were treated for 1 h with chlorpromazine, dynasore or pitstop 2. Then, they were maintained for 30 min on ice with Tf CF^®^543 (5 μg/mL). Finally, cells were incubated 5 min at 37 °C before fixation. (**C**) Quantification of Tf conjugate. ROIs from groups of 20 cells and three areas of each image were measured. Measurement of mean fluorescence intensity from the 555 channel in an ROI was performed. The mean percentage of fluorescence ± S.D. is shown. (*n* = 4); * *p* < 0.05. (**D**) The uptake of dextran in the Huh-7 cells was not disrupted by the CME inhibitors. Cells were treated for 1 h with chlorpromazine, dynasore or pitstop 2. Then, they were maintained for 30 min on ice with CF^®^555 Labeled Dye Dextran 10,000 MW (5 μg/mL). Finally, the cells were incubated for 10 min at 37 °C before fixation. (**E**) Quantification of dextran conjugate. Same procedure as with Tf quantification. Scale bar = 20 µm. * *p* < 0.05.

All these drugs were first subjected to a MTT assay to study their potential cytotoxicity in Huh-7 cells, the experimental model chosen for these assays, as they have been previously reported to be highly susceptible to HCoV-229E infection [17]. After 24 h of incubation with drugs, the cells treated with 10 µM chlorpromazine, 100 µM dynasore or 50 µM pitstop 2 (Figure 1A) maintained viability above 70%, which is the threshold recommended by the ISO 10993-5:2009 (E) standard [31]. These concentrations were chosen for the rest of the assays. 

Human transferrin is known to use the CME route for cell entry [6]. In this study, human transferrin conjugate Tf CF^®^543 was chosen to establish a reliable method for monitoring CME endocytosis in Huh-7 cells and to demonstrate that the CME-inhibitory drugs used effectively block this pathway, as we previously performed in previous works in other cell lines [32].

Fluorescence microscopy images showed a decrease in Tf conjugate uptake in drug-treated cells compared to the non-treated samples (Figure 1B), results supported by the quantification of this uptake (Figure 1C). The drugs then reduced Tf internalization, suggesting a partial blockade of CME. Notably, the dynasore- and pitstop 2-treated cells exhibited no appreciable Tf uptake, whereas the chlorpromazine-treated cells showed minor blocking of Tf uptake. However, this uptake was significantly lower compared to the non-treated cells. In particular, the dynasore exhibited a dose-dependent inhibition of Tf uptake, with the highest non-cytotoxic concentration producing the strongest inhibitory effect (Figure S1A). As CME-inhibitory drugs are criticized for reported indirect effects [33], an analysis of the uptake of dextran conjugate, which is not internalized by CME but by other independent routes, was performed. At the concentrations tested, the dextran uptake was not altered (Figure 1D,E) while the Tf internalization was blocked.

### 3.2. Effect of CME by Inhibitory Drugs in HCoV-229E Infection

**Figure 2:**
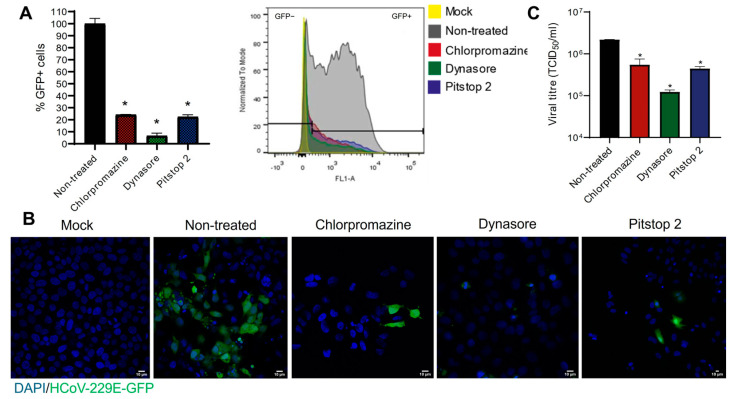
HCoV-229E infection is blocked by CME-inhibitory drugs. Huh-7 cells were treated with either 10 µM chlorpromazine, 100 µM dynasore or 50 µM pitstop 2 for 1 h and subsequently infected with HCoV-229E-GFP at an m.o.i. of 0.5. Cells were maintained in the presence of the drugs until they were collected at 20 h p.i. (**A**) Flow cytometry analysis: this graph shows the percentage of normalized infection at 20 h p.i. ± S.D. Triplicate experiments were performed (*n* = 4). (**B**) The fluorescence microscopy images show the GFP+ signal, which corresponds to viral infection. Cellular nuclei are stained with DAPI. Scale bar = 10 µm. (**C**) Infectious particles of the progeny virus were titrated in Huh-7 cells to determine the 50% tissue culture infective dose (TCID_50_)/mL. The graph shows the mean ± S.D. (*n* = 3) viral production. * *p* < 0.05.

After validating the desired inhibitory effect and concentration of drugs for the rest of this study, the following step was to analyze the effect of such drugs in the entry and infection of HCoV-229E. Briefly, cells were treated with either chlorpromazine, dynasore or pitstop 2 for 1 h, and then they were infected with the virus. The cells were maintained in the presence of the drugs during and after the infection, and samples were collected at 20 h p.i. for flow cytometry and immunofluorescence. When treated with the drugs, a significant decrease in the infection was reported as compared to non-treated cells, as seen in the flow cytometry analysis (Figure 2A), immunofluorescence images (Figure 2B), showing GFP corresponding to viral infection, and titration of viral infectious particles (Figure 2C). The inhibition of the infection in the CME-treated cells, in particular, those treated with dynasore, was achieved in a dose-dependent manner, with 100 µM being the concentration that showed the greatest reduction in infection (Figure S1B). The inhibition of CME by using pharmacological drugs reduces HCoV-229E infection, and thus, the virus might be using this route to enter Huh-7 cells.

### 3.3. Deletion of AP2M1 Reduces HCoV-229E Infection

**Figure 3:**
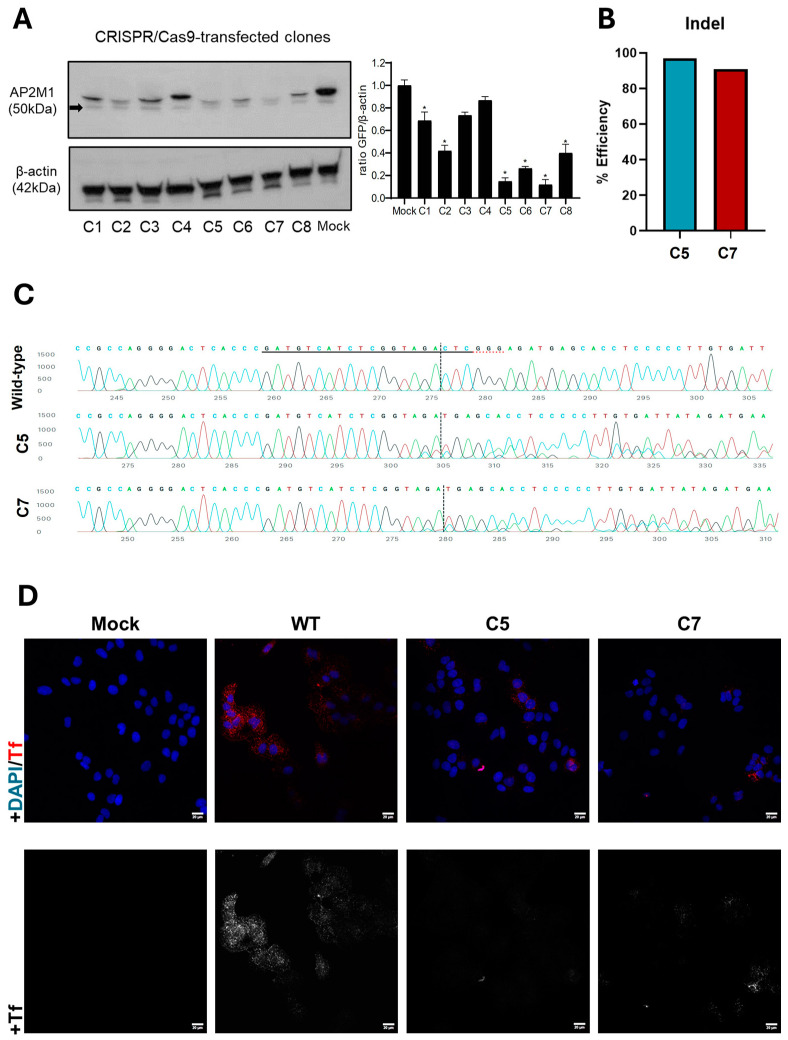
Validation of the knockout of the AP2M1 gene in Huh-7 cells by CRISPR/Cas9. (**A**) AP2M1 expression was checked by immunoblotting. Western blot analysis of total cell lysates subjected to SDS-PAGE, showing AP2M1 for each subcloned cell. β-actin was chosen as the protein loading control. The black arrow points to the specific AP2M1 band. Values of immunoblot quantification are reported as mean ± S.D. (*n* = 3); * *p* < 0.05. (**B**) Indel values of C5 and C7. Percentage of efficiency of CRISPR-Cas9 cut. (**C**) Sanger sequence view showing wild-type (control) and edited (C5, C7) sequences in the region around the guide sequence. The horizontal black underlined region represents the guide sequence. The horizontal red underline is the PAM site. The vertical black dotted line represents the actual cut site. Cutting and error-prone repair usually result in mixed sequencing bases after the cut. (**D**) Conjugate transferrin internalization is blocked in AP2M1-KO cells. The uptake of transferrin in KO AP2M1 Huh-7 cells C5 and C7 is blocked. Cells were maintained for 30 min on ice with Tf CF^®^543 (5 μg/mL). Finally, they were incubated 5 min at 37 °C before fixation. Nuclei were stained with DAPI. Scale bar = 20 µm. Original images can be found in Supplementary Materials.

To analyze the impact of CME in HCoV-229E infection, a loss-of-function assay was performed to inhibit AP-2, an adaptor that plays an essential role in this route [34]. The CRISPR/Cas9 system was chosen to knock out the AP2M1 gene in the Huh-7 cell line, coding for subunit µ of the adaptor. After the subcloning of the KO pool, the absence of AP2M1 protein expression was analyzed by immunoblotting (Figure 3A). Furthermore, amplification of the sequencing of the targeted zone was performed, and the high percentage of indels (Figure 3B) suggests that the CRISPR-Cas9 system has been effective in targeting and cutting the specific DNA sequence, especially in the KO cells named C5 and C7. The Sanger sequences (Figure 3C) also report that CRISPR-Cas9 was successfully cut, altering the reading frame. The sequences present in the edited population and their relative proportions were analyzed too, discarding those that showed more than three different sequences and an indel number lower than 90% (Figure S2). In addition, the viability and morphology of the C5 and C7 KO cells were studied, revealing no differences from the wild-type Huh-7 cells (Figure S3A,B). The internalization of the conjugate Tf uptake was also studied in AP2M1 KO cells by immunofluorescence, revealing that when the subunit µ of AP-2 is not present, the CME route is disrupted and Tf does not enter at the same levels as in wild-type Huh-7 (Figure 3D).

**Figure 4:**
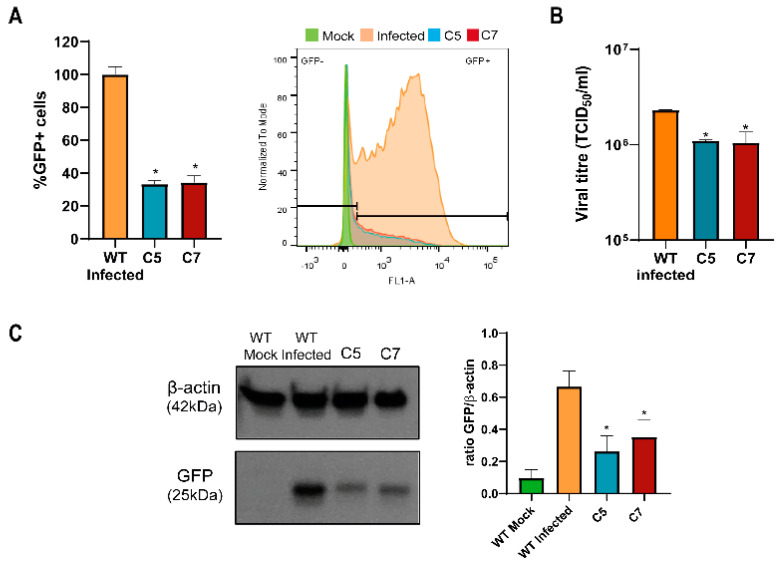
The knockout of AP2M1 in Huh-7 cells contributed to the decrease in HCoV-229E infection. KO cells C5 and C7 and non-KO cells (wild-type) were infected with HCoV-229E-GFP at an m.o.i. of 0.5. At 20 h p.i., samples were processed, and infection was analyzed by the following techniques: (**A**) Flow cytometry data show the mean of the percentage of normalized infection 20 h p.i. (% GFP+ cells) ± S.D. (*n* = 4). * *p* < 0.05. The plots represent the histograms of GFP-positive (+) and GFP-negative (−) cells. (**B**) Infectious particles of the progeny virus for each condition were titrated in Huh-7 cells to determine the 50% tissue culture infective dose (TCID_50_)/mL. The graph shows the mean ± S.D. (*n* = 3) viral production. (**C**) Western blot analysis of total cell lysates subjected to SDS-PAGE, showing the viral GFP for each condition. β-actin was chosen as the protein loading control. Values of immunoblot quantification are reported as mean ± S.D. (*n* = 3); * *p* < 0.05. Original images can be found in Supplementary Materials.

Once the AP2M1 KOs were validated, the infection of C5 and C7 with HCoV-229E-GFP was performed. Compared to the wild-type non-KO cells, the infection of cells that did not express AP2M1 was reduced significantly, more than 50%, as seen in the flow cytometry data (Figure 4A), viral titration (Figure 4B) and immunoblot (Figure 4C). When AP-2 does not recruit clathrin, the infection of HCoV-229E is reduced, suggesting that CME plays an important role in the entry of this virus to the Huh-7 cell line.

### 3.4. HCoV-229E Uses the Endosomal Pathway and Needs Low pH to Enter the Cell

**Figure 5:**
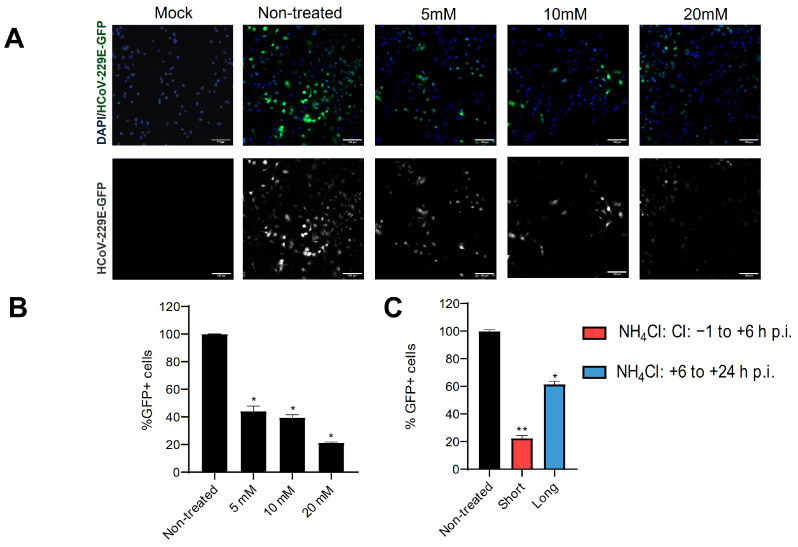
Inhibition of endosomal acidification prevents the entry of HCoV-229E in the Huh-7 cell line. Huh-7 cells were treated with NH_4_Cl at different concentrations for 1 h and during infection with HCoV-229E-GFP at an m.o.i. of 3. (**A**) Fluorescence microscopy images showing GFP-positive cells at 20 h p.i. and nuclei stained with DAPI for each condition. Scale bar = 100 µm. (**B**) The flow cytometry data report the mean percentage of GFP+ cells, normalized to the non-treated control. * *p* < 0.05 (*n* = 4). (**C**) Huh-7 cells were treated with 20 mM NH_4_Cl from 1 h before infection to 6 h p.i. (short times) or from 6 to 24 h p.i. (long times), with HCoV-229E-GFP at an m.o.i. of 3. The flow cytometry analysis reports the mean percentage of GFP+ cells at 24 h p.i., normalized to the non-treated control. * *p* < 0.05; ** *p* < 0.01 (*n* = 4).

Previous research reports that in other cell lines, HCoV-229E needs an acidic environment to utilize the endosomal pathway [9,12,21]. Consistent with previous data, the treatment of Huh-7 cells with NH_4_Cl, a lysomotropic agent that prevents the acidification of the endosome [35], demonstrated a contribution of low pH to the infection. The results show that, when treated with NH_4_Cl, the infection was inhibited in a dose-dependent manner (Figure 5A,B). In addition, when NH_4_Cl was added at early times (−1 to +6 h p.i.), viral titration strongly decreased (Figure 5C), suggesting that the acidification of the endosomes could be critical during cell entry. In contrast, the infection barely decreased when NH_4_Cl was added at 6 h p.i, confirming that the agent has no effect on the post-fusion step of the viral cycle. Therefore, early HCoV-229E infection in Huh-7 is also sensitive to endosomal pH neutralization, requiring acidification of the endosomal compartment.

**Figure 6:**
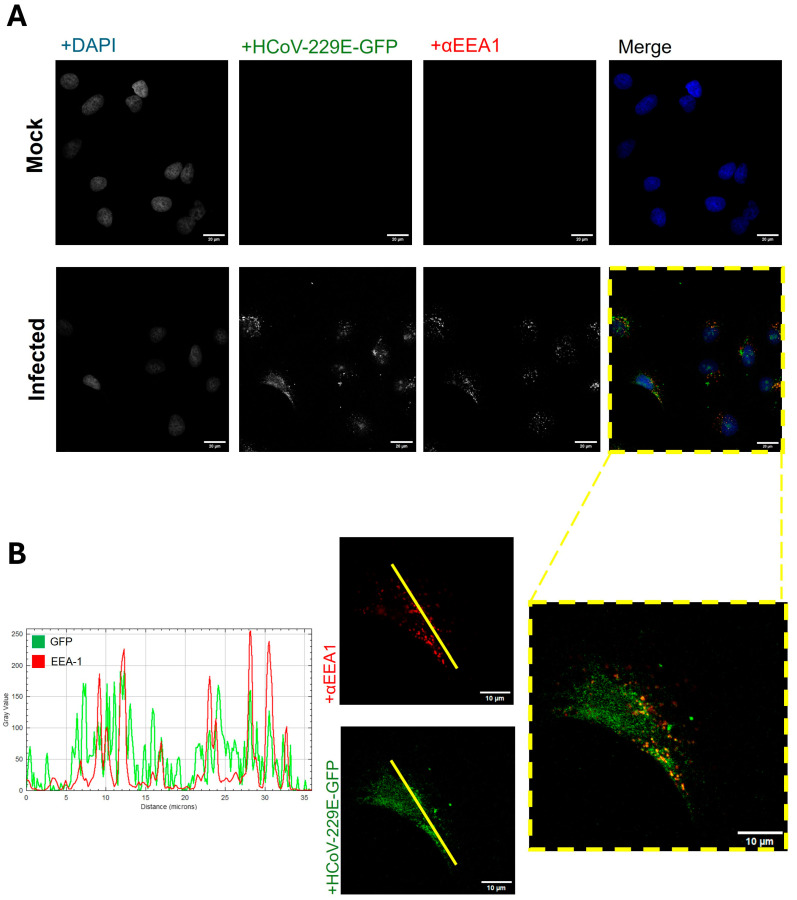
Quantitative confocal analysis of the colocalization of HCoV-229E and EEA-1 signal hot spots. (**A**) Cells were maintained with HCoV-229E for 15 min on ice and then incubated for 30 min at 33 °C. Then, they were fixed and labeled with the EEA-1 primary antibody and AlexaFluor-555-conjugated secondary antibody (red). The nuclei were stained with DAPI (blue). In the far-right panel, all three signal bandwidth images are combined, with colocalized red and green signals seen as yellow. Scale bar = 20 µm. (**B**) Two lineal sections were selected for each signal (yellow lines). The red EEA-1 and green viral GFP signal intensities in this line were plotted as a function of X–Y distance across the cell. Scale bar = 10 µm.

Furthermore, to provide further confirmation that HCoV-229E uses the endocytic pathway in Huh-7 cells, dual immunofluorescence labeling with antibodies specific for the HCoV-229E N protein and early endosome antigen 1 (EEA-1), located exclusively in the early endosomes, was performed (Figure 6A). Confocal microscopy analyses revealed a strong correlation between the signal intensities of HCoV-229E N and EEA-1 (Figure 6B), not only seen in the merged signals but also in the relative fluorescence intensity peaks.

## 4. Discussion

The entry of enveloped viruses is described to occur through two primary pathways: (i) direct entry via fusion of the viral envelope with the host cell plasma membrane, with consecutive genome delivery to the cytosol, and/or (ii) use of the cell’s endocytic machinery, where a low-pH environment and clathrin-mediated endocytosis may play important roles [5,36]. Although infection mechanisms have been thoroughly studied for other coronaviruses such as SARS-CoV-2, there remains a lack of understanding regarding catarrhal HCoVs and their impact on host cells. Additionally, since these viruses have the potential for zoonotic transmission and co-infection, which could lead to the creation of new recombinant strains, they are especially relevant in the context of future pandemics [37].

HCoV-229E has already been reported to use caveolae-mediated endocytosis to enter L-132 cells and human fibroblasts [12]. In addition, in that research, no clathrin-coated pits were observed by electron microscopy analysis in the vicinity of viral particles. Nevertheless, it should be noted that CME is primarily receptor-mediated and tumor liver tissue (Huh-7 cells come from hepatocarcinoma cells) is largely more positive for APN expression than fibroblasts [3]. Previous studies have reported the internalization of the main receptor of HCoV-229E, APN, through a clathrin-dependent mechanism in polarized hepatic cells [38]. In the HT1080 cell line, when the RECK protein is absent, APN is mainly internalized along with the markers of CME and caveolae endocytosis [39]. Thus, once HCoV-229E has anchored to APN, both may follow CME. Related to this, the human hepatoma cell line Huh-7 was the experimental model of choice, as it has been previously reported to be highly susceptible to HCoV-229E infection [17]. In addition, Huh-7 cells are polarized epithelial cells that highly express APN, and the entry and release of many HCoVs, including HCoV-229E, have been previously associated with the apical cells [18,19].

This is the first time that a study has suggested that HCoV-229E enters human liver epithelial cells through a clathrin-dependent route. The decrease in the infection when blocking CME with dynasore, chlorpromazine and pitstop 2 supports the hypothesis that HCoV-229E is using this route to enter and infect Huh-7 cells. In addition, the low infection rates when infecting AP2M1 KO cells provide further evidence. AP-2, the second most abundant component of the clathrin vesicles, is crucial for clathrin-coated pit maturation. Without AP-2, clathrin cannot be recruited and mature vesicles will fail to form [20]. AP-2 is a heterotetramer of the α, β, σ and μ subunits. Phosphorylation of the μ subunit by AP-2 associated protein kinase 1 enhances clathrin interaction and cell surface receptor incorporation [40]. Several studies have shown that the μ subunit acts as a physical linker between clathrin triskelions and internalization sequences in receptors on the cell membrane during the initial step of clathrin-coated vesicle (CCV) formation. AP-2 is organized into two heterodimers: α/σ and β/μ. The absence of the μ subunit disrupts the assembly of the β/μ heterodimer, leading to the degradation of the β subunit. Consequently, the lack of a sufficient β/μ heterodimer prevents full AP-2 assembly, which in turn induces the degradation of the α/σ heterodimer [41]. As there are no chemical inhibitors for AP-2, we used the CRISPR/Cas9 strategy to deplete the μ subunit, disrupting AP-2 assembly and causing the degradation of both heterodimers, thus preventing clathrin-coated pits’ formation [41]. Previous studies in our group have made knock-downs of AP2M1 by previously defined shRNA pools, demonstrating that depletion of AP-2 does not affect cellular viability [32] but CRISPR/Cas9 is a better alternative to assure a complete depletion of the gene.

Furthermore, the importance of low-pH conditions was also demonstrated. Supporting our results, previous publications have reported the relationship between HCoV-229E virions and the endosomal route [9,22,42]. As shown, the virus may follow the endocytic route by colocalizing with the EEA-1 marker, only present in early endosomes, and requiring a low pH for productive infection, as viral infection dropped during treating with NH_4_Cl at early times. It should be noted that acidification of the endosomal environment is required for successful fusion and release of the viral genome into the cytoplasm [43]. In detail, virion release of HCoV-229E is thought to be dependent on some host factors, such as valosin-containing protein, while other factors, such as inducible transmembrane proteins, may impede the uncoating of some RNA viruses [44]. Other reports also suggested that this valosin-containing protein is involved in the maturation of early endosomes and decomposition of the nucleocapsid of HCoV-229E in the early steps of the infection [45].

This study contributes to previous information about the mechanisms of infection of HCoV-229E, especially in the entry phase of the viral cycle. Understanding the internalization mechanisms of this virus can lead to well-designed treatments against viral particles using CME to enter the cell. However, further research must be performed to see if this route is maintained from in vitro to in vivo studies. Especially in vivo, studies on CME cargo are challenging due to the large quantity of different cargoes undergoing endocytosis at the same time [4]. In addition, it is evident that endocytosis will exhibit even greater variability among different cell types in vivo, reflecting the unique characteristics of each cell, their physiological roles and the constantly shifting local environments of cells in various tissues throughout an organism [46]. It has been demonstrated that the virus uses host-cell CME to enter Huh-7 cells, as suggested by AP2M1 KOs and pharmacological treatment with inhibitory drugs. In addition, this process requires a functional early endosomal compartment and depends on endosomal acidification to achieve productive infection, as seen in other cell lines [9,22]. However, the study of other mechanisms of entry should not be discarded, bearing in mind that CME and endosomes are important but not the only routes of entry [12]. As mentioned, further research, particularly in primary cell cultures, would be necessary to keep distinguishing the intracellular trafficking route and the extent to which clathrin and AP-2 are involved in the entry route of HCoV-229E.

## 5. Conclusions

In conclusion, this study reveals that HCoV-229E enters Huh-7 cell line via clathrin-mediated endocytosis (CME), as supported by two main approaches: (i) CME-inhibitory drugs that reveal a decrease in viral entry and (ii) knock-out of AP2M1, with AP-2 being a key component in the recruitment of clathrin, and KO-cells being less susceptible to infection. The virus also depends on low-pH endosomal environments for infection, confirmed by reduced viral activity in alkaline conditions. While CME is confirmed as a primary entry mechanism in Huh-7 cells, other entry pathways may exist. Further in vivo research is needed to fully understand these mechanisms.

## Data Availability

The data presented in this study are available on request from the corresponding author upon reasonable request.

## Supplementary Materials

The following supporting information can be downloaded at: https://www.mdpi.com/article/10.3390/biom14101232/s1, Figure S1: Dose dependent effect of the CME blocking drug dynasore in Huh-7 cells; Figure S2: Sequencing of subcloned *AP2M1* KO pool and relative contribution of sequences; Figure S3: Morphology of AP2M1-KO Huh-7 cells; Figure S4: Original western blot images from (A) Figure 3 and (B) Figure 4. Original images from Western blot can be found in Supplementary Materials.
