# Supplementary material for: Human Coronavirus 229E Uses Clathrin-Mediated Endocytosis as a Route of Entry in Huh-7 Cells
Source: Biomolecules. 2024 Sep 29;14(10):1232. doi: 10.3390/biom14101232 (PMC11505773; doi:10.3390/biom14101232)
Supplement: Supplementary file 1 [file biomolecules-14-01232-s001.zip › biomolecules-3171772-supplementary.pdf]

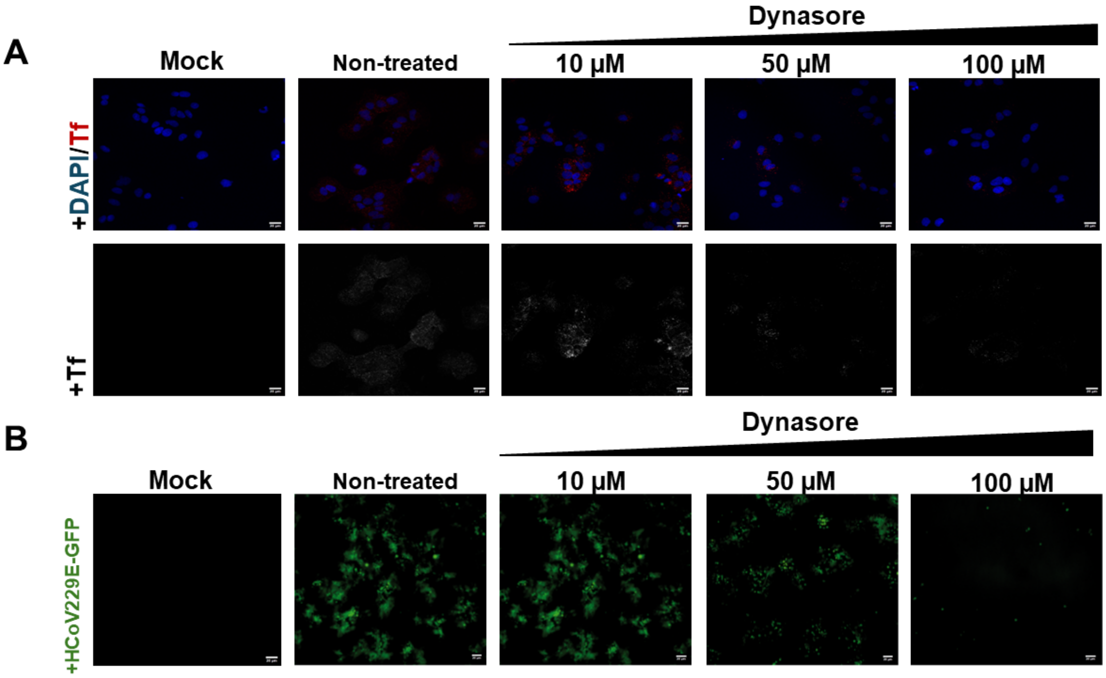

Figure S1. Dose dependent effect of the CME blocking drug dynasore in Huh-7 cells. A) The uptake of Tf cells is blocked by dynasore. Cells were treated for 1 h with dynasore at different non-cytotoxic concentrations. Then, they were maintained for 30 min on ice with Tf CF®543 (5 µg/mL). Finally, cells were incubated 5 min at 37°C before fixation. Nuclei are stained with DAPI. Scale = 20 µm **B) HCoV-229E infection is blocked when treating with dynasore.** Cells were treated with increasing concentrations of dynasore for 1 h and subsequently infected with HCoV-229E-GFP at an m.o.i of 0.5. Cells were maintained in the presence of the drugs until they were collected at 20 h p.i.. Fluorescence microscopy images show GFP+ signal which corresponds to viral infection. Scale = 20 µm

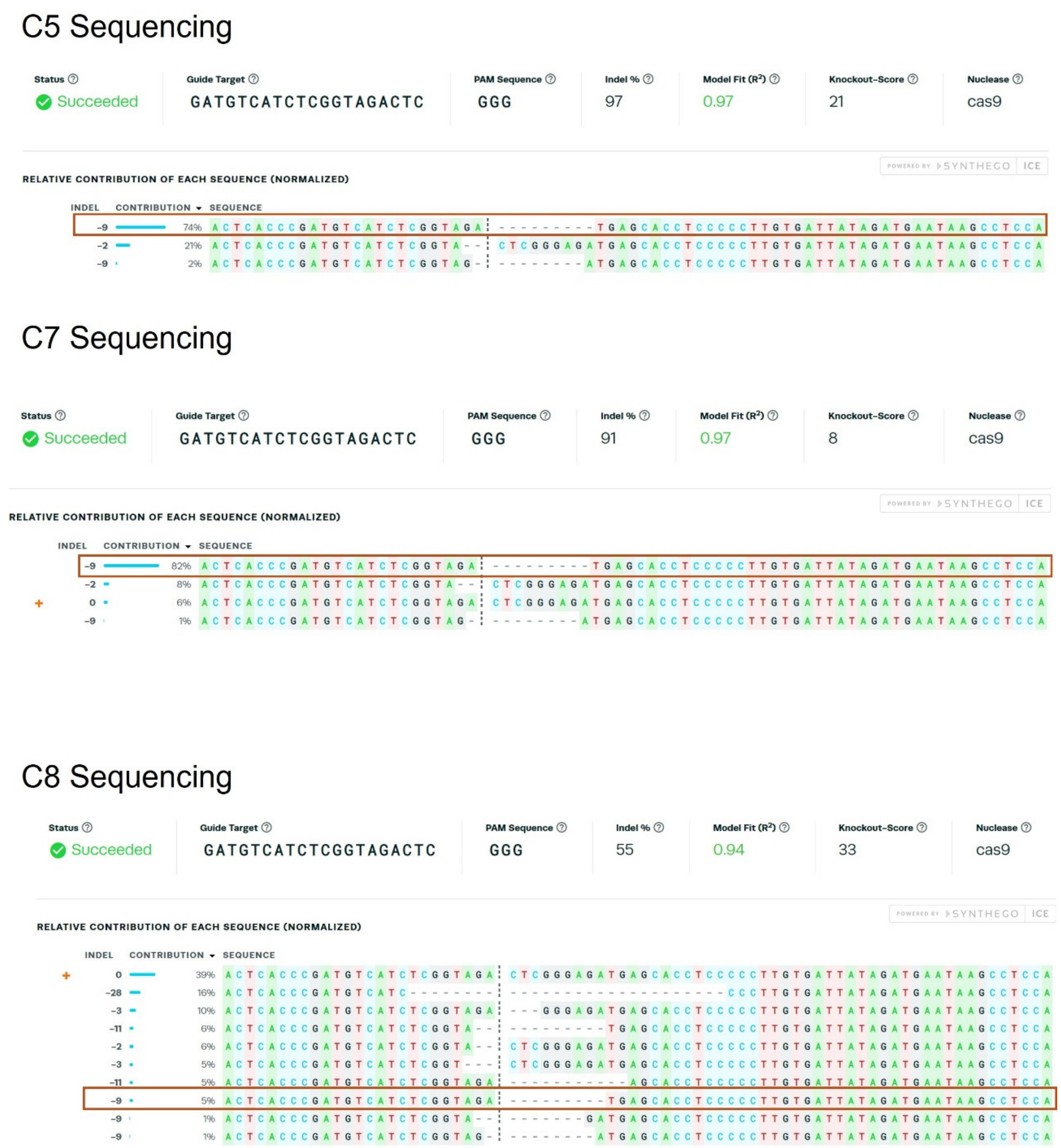

Figure S2. Sequencing of subcloned *AP2M1* KO pool and relative contribution of sequences. The contributions show the normalized inferred sequences present in the edited population and their relative proportions. Cut sites are represented by black vertical dotted lines, and the wild-type sequence is marked by a "+" symbol on the far left.

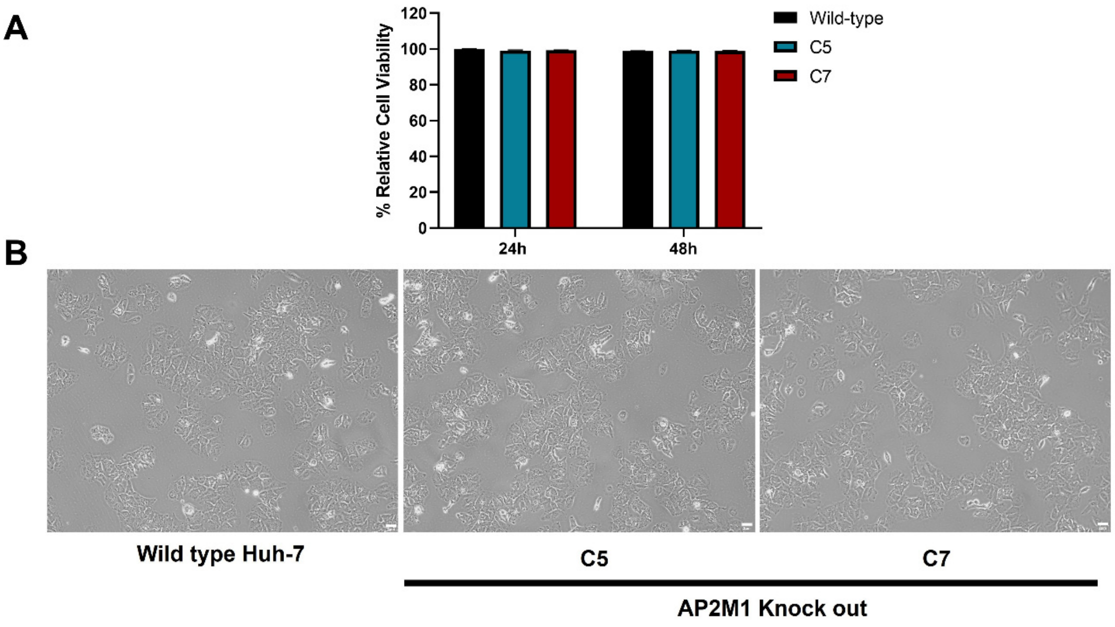

Figure S3. Morphology of AP2M1-KO Huh-7 cells. A) Cell viability of wild-type Huh-7, and AP2M1-KO cells C5 and C7. Viability was measured by MTT tetrazolium salt assay and represented as the mean percentage of cellular viability ± S.D. (n = 4) after 48h. B) Brightfield microscopy images of wild-type Huh-7, and AP2M1-KO cells C5 and C7. There are no observable differences in size, shape, or volume between them.

A

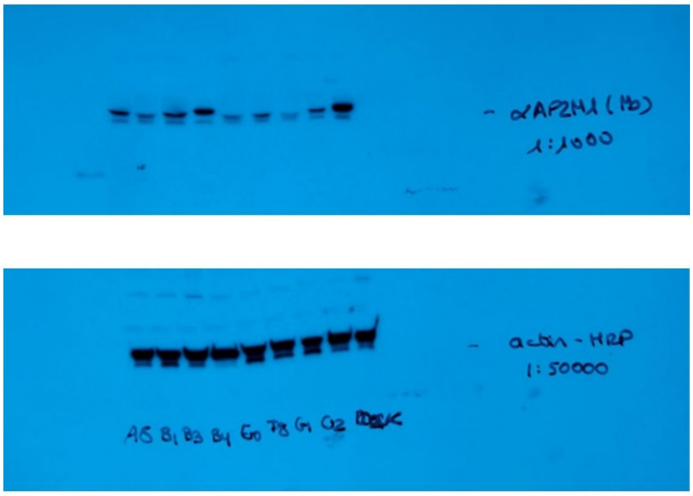

B

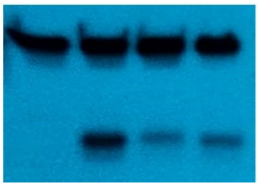

Figure S4. Original western blot images from A) Figure 3 and B) Figure 4.
